# X-ray micro-computed tomography reveals a unique morphology in a new click-beetle (Coleoptera, Elateridae) from the Eocene Baltic amber

**DOI:** 10.1038/s41598-020-76908-3

**Published:** 2020-11-19

**Authors:** Robin Kundrata, Andris Bukejs, Alexander S. Prosvirov, Johana Hoffmannova

**Affiliations:** 1 Department of Zoology, Faculty of Science, Palacky University, 17. listopadu 50, 771 46 Olomouc, Czech Republic; 2 Institute of Life Sciences and Technologies, Daugavpils University, Vienības 13, Daugavpils, 5401 Latvia; 3 Department of Entomology, Faculty of Biology, Moscow State University, Leninskie gory 1/12, Moscow, Russia 119234

**Keywords:** Zoology, Entomology, Palaeontology, Taxonomy

## Abstract

Beetle fossils are a rich source of information about the palaeodiversity and evolutionary history of the order Coleoptera. Despite the increasing rate of fossil research on click-beetles (Coleoptera: Elateridae), the most diverse group in the superfamily Elateroidea, their fossil record has remained largely unstudied. This may be caused by the combination of their rather uniform external morphology and the suboptimal state of preservation and visibility in most fossil specimens. Here, we used X-ray micro-computed tomography to reconstruct the morphology of an interesting click-beetle from Eocene Baltic amber, which had some principal diagnostic characters obscured by opaque bubbles and body position. Our results suggest that the newly described *Baltelater bipectinatus* gen. et sp. nov. belongs to tribe Protelaterini within subfamily Lissominae. Since Protelaterini have a predominantly Gondwanan distribution, our discovery is of a great importance for the historical biogeography of the group. Very distinctive are the bipectinate antennae with 11 antennomeres and with rami beginning on antennomere IV, which are not found in any recent Elateridae. The discovery of a new click-beetle lineage from European Eocene amber sheds further light on the palaeodiversity and historical diversification of the family as well as on the composition of the extinct amber forest ecosystem.

## Introduction

Over recent decades, there has been an increasing amount of research attention directed towards insect fossils. This led to the discoveries of various previously unknown lineages, and helped to improve our knowledge on the origin, evolution, palaeodiversity and palaeobiogeography of insect groups at all taxonomic levels^1–5^. Among fossils, amber inclusions play an important role because they represent the finest and most complete insect fossil remains due to their three-dimensional nature, and are relatively easily compared with extant forms^6–13^. However, amber fossils are often in a bad state of preservation that does not enable the visualization of crucial morphological features^14,15^. In order to overcome this problem, micro-computed tomography can be employed. This non-destructive technology allows the effective reconstruction of fossilized structures in three dimensions using digital software even for suboptimally preserved specimens^16^. Recently, micro-CT data were used in palaeontological studies focused on the morphology of various fossil animal taxa, including, for example, arachnids^17,18^, myriapods^19^, crustaceans^20^, insects^3,21^, and also vertebrates^22,23^. This method is used not only for habitus observation but often also for reconstruction of internal organs including genitalia, as documented by a number of recent papers dealing especially with Baltic amber Coleoptera^24–27^.

Baltic amber constitutes the largest known Paleogene source of fossilized plant resin worldwide, and contains the most diverse assemblage of fossil insects to date^2,28,29^. Although the inclusions in Baltic amber are well-known for their preservation in exquisite detail, and the number of publications on these fossils has been rapidly growing, there have been uncertainties regarding the geographic origin, botanical provenance, and age of this amber. Baltic amber is not found in its original stratigraphic position; instead, it has been redeposited mainly in marine sediments and fluvial deposits. It occurs in the "Blue Earth" layers of the Baltic region, especially in the Samland Peninsula within the Kaliningrad Oblast (Russia), but also in other countries along the coast of the Baltic Sea, including Denmark, Germany, Poland, Sweden, and the Baltic states^29^. The original habitat of the amber forest has been assumed to be a thermophilic, humid mixed forest^30^ in a warm temperate, humid, equable climate without thermal seasonality^11^. There has been conflicting evidence about the source plant of this amber, and this was officialy termed the "Tertiary Baltic amber mystery"^31^. For many years it was believed that *Pinus succinifera* (Göppert) Conwentz was the main resin producer^6,32^; however, more recent research brought more candidates, including the representatives of Sciadopityaceae, Araucariaceae, Pinaceae, and Cupressaceae^8,33–35^. The age of Baltic amber has also been the subject of much debate, usually due to the repeated re-deposition of the amber, the broad range of the ancient resin-producing forest, and its probable long-term existence^36^. Using various methods such as K–Ar dating, palynological biostratigraphic analysis or comparison of the faunas from different amber sources, most estimates generally range from the middle to upper Eocene (Lutetian to Priabonian, 47.8–37.8 Ma)^28,36–40^.

Elateridae, or click-beetles, is the largest family in the polyphagan superfamily Elateroidea. Its typical representatives are well-known for their hard, compact body and the pro-mesothoracic clicking mechanism^41,42^; however, there are several soft-bodied lineages currently included in the family which were historically considered separate families^43–45^. The composition, phylogeny and natural classification of Elateridae are far from fully understood, despite the effort of numerous recent studies^43,44,46,47^. Elateridae comprise more than 10,000 extant species worldwide^42^, and the fossil record includes approximately 300 species, although the placement of many of them needs thorough investigation^48,49^. They originated and greatly diversified during the Mesozoic^2,50,51^, and are among the most common beetle families recorded in Baltic amber^28,52^. Until now, 16 click-beetle species have been described from Baltic amber, with one classified in Agrypninae, one in Cardiophorinae, two in Dendrometrinae, 10 in Elaterinae, and one *incertae sedis*^51,53,54^. Additionally, representatives of more than 20 genera have been reported from Baltic amber without detailed descriptions^28,52,55–59^, and many yet unstudied specimens are waiting to be examined in various collections.

**Figure 1:**
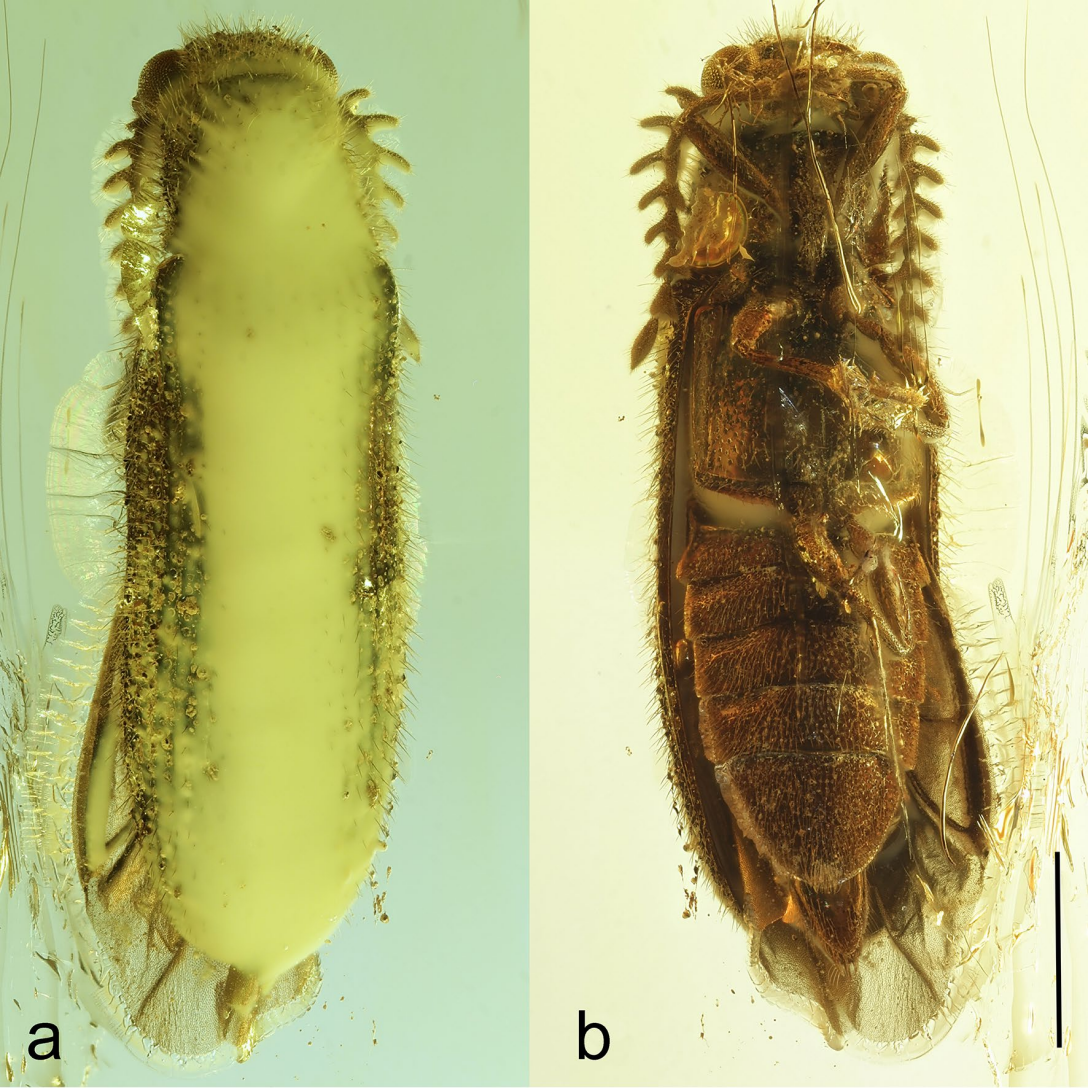
Habitus of *Baltelater bipectinatus* gen. et sp. nov., holotype: (**a**) dorsal view; (**b**) ventral view. Scale bar = 1.0 mm. Credit: Jonas Damzen (Vilnius, Lithuania).

In this study, we use X-ray micro-computed tomography (μCT) to reconstruct the morphology of a Baltic-amber click-beetle specimen which has some principal diagnostic characters obscured by opaque bubbles and suboptimal body position (Fig. 1). Results of our investigation suggest that the studied specimen represents a new genus and species in the tribe Protelaterini within the subfamily Lissominae. The discovery of the first lissomine click-beetle from European Eocene amber sheds further light on the palaeodiversity and historical diversification of the family as well as on the composition of the extinct amber forest ecosystem.

## Results

**Figure 2:**
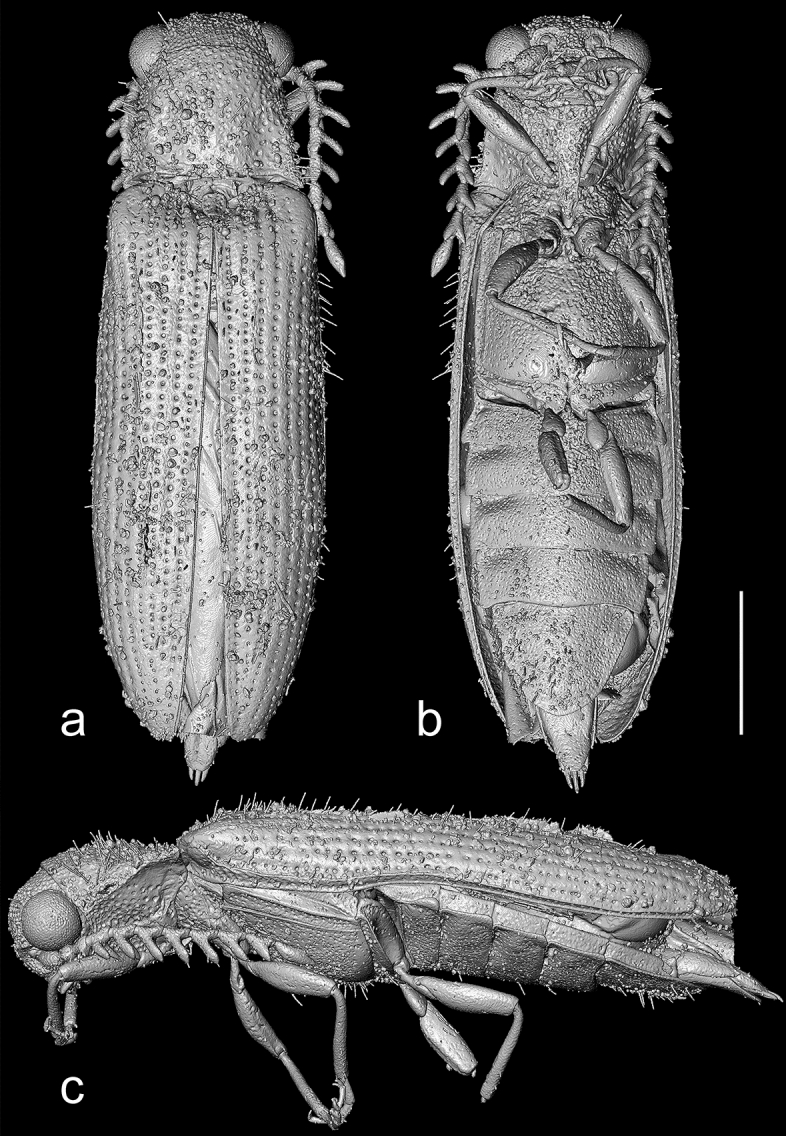
X-ray micro-CT renderings of *Baltelater bipectinatus* gen. et sp. nov., holotype, habitus: (**a**) dorsal view; (**b**) ventral view; (**c**) left lateral view. Scale bar = 1.0 mm.

**Figure 3:**
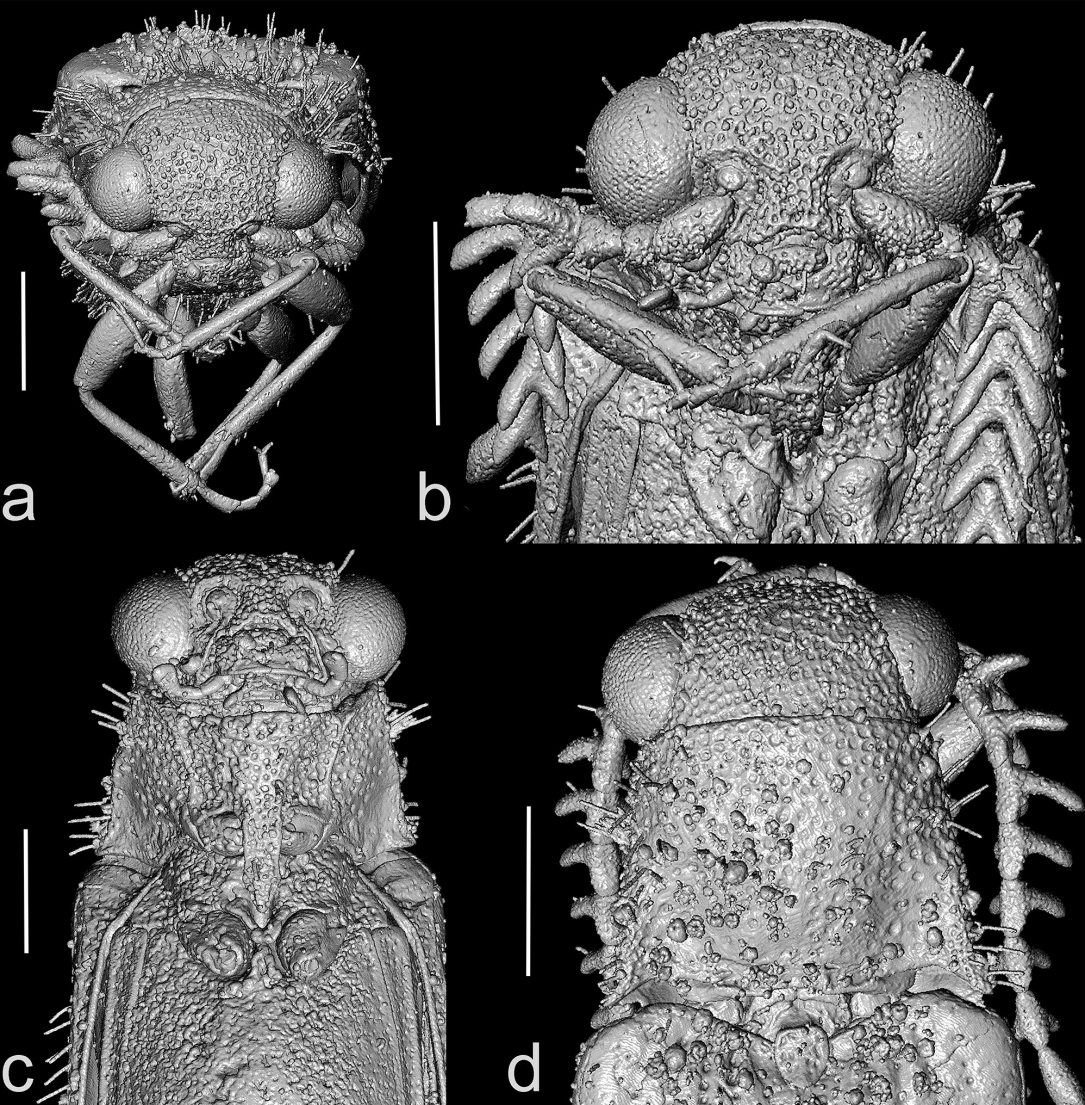
X-ray micro-CT renderings of *Baltelater bipectinatus* gen. et sp. nov., holotype: (**a**) head, frontal view; (**b**) head, frontoventral view; (**c**) head and thorax (legs removed), ventral view; (**d**) head and thorax, dorsal view. Scale bars = 0.5 mm.

**Figure 4:**
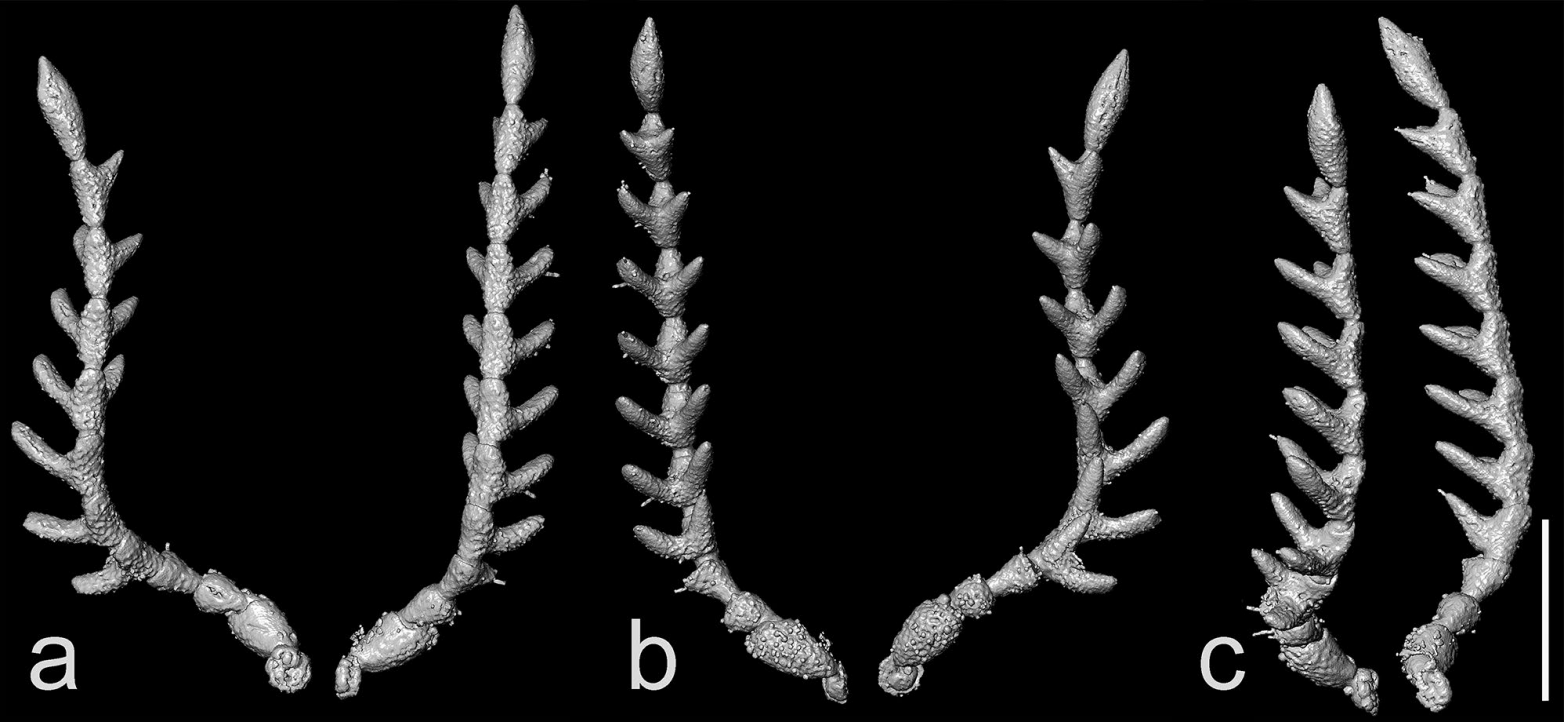
X-ray micro-CT renderings of *Baltelater bipectinatus* gen. et sp. nov., holotype, antennae: (**a**) dorsal view; (**b**) ventral view; (**c**) lateral view. Scale bar = 0.5 mm.

**Figure 5:**
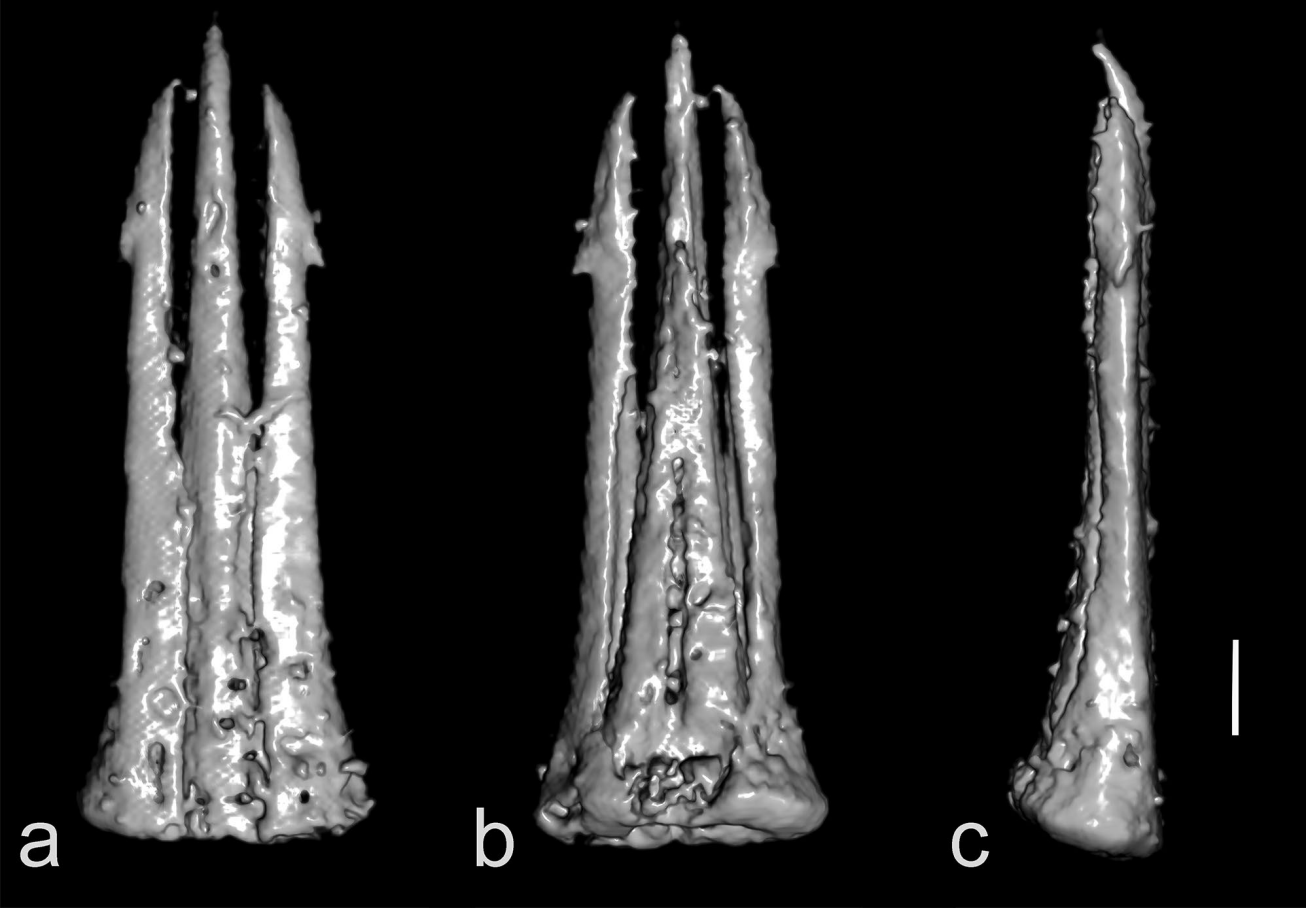
X-ray micro-CT renderings of *Baltelater bipectinatus* gen. et sp. nov., holotype, aedeagus (without phallobase): (**a**) dorsal view; (**b**) ventral view; (**c**) lateral view. Scale bar = 0.1 mm.

Based on its divergent morphology (Figs. 1–5, Supplementary Videos 1–3), we describe here *Baltelater bipectinatus* gen. et sp. nov., which we classify in the tribe Protelaterini within the click-beetle subfamily Lissominae.

### Systematic paleontology

Family Elateridae Leach, 1815

Subfamily Lissominae Laporte, 1835

Tribe Protelaterini Schwarz, 1902

 = Senodoniini Schenkling, 1927

 = Sphaenelaterini Stibick, 1979

*Type genus*. *Protelater* Sharp, 1877.

*Diagnosis*. Body elongate, oblong, rather convex; scape more or less straight and moderately long; prothorax and elytra not tightly joined together, narrowed at junction of prothorax and elytra; prosternal chin-piece short and usually truncate; hypomeron without cavities for reception of antennae; pro- and mesotrochanters less than 2.5 times as long as wide; tibia subcylindrical and not compressed laterally; tarsomeres I–IV each with membranous lamella apico-ventrally (these lamellae less developed especially on tarsomeres I and II); styli of ovipositor present, small, attached subapically or medially.

*Composition and distribution*. 47 described species classified in nine recent genera, i.e., *Anaspasis* Candèze, 1882 (4 spp., South America), *Austrelater* Calder & Lawrence, 1993 (3 spp., Australia), *Protelater* Sharp, 1877 (12 spp., New Zealand), *Rostricephalus* Fleutiaux, 1918 (1 sp., East and South East Asia), *Senodonia* Laporte, 1838 (20 spp., South, East and South East Asia), *Sossor* Candèze, 1883 (1 sp., South East Asia), *Sphaenelater* Schwarz, 1902 (3 spp., New Zealand), *Tunon* Arias-Bohart, 2013 (1 sp., South America), *Valdivelater* Lawrence & Arias, 2009 (2 spp., South America)^47,60,61^, and one monotypic fossil genus, i.e., *Baltelater* gen. nov. (North European Baltic amber).

### *Baltelater* gen. nov

urn:lsid:zoobank.org:act:A0AD4F5E-2B55-4731-8B40-F4A0470DA29A.

*Type species*. *Baltelater bipectinatus* sp. nov.; by present designation.

*Diagnosis*. Adult male. Head globular, deflexed; eye large, prominent, entirely visible, mouthparts hypognathous; mandible rather broad and short, bidentate; frons slightly overhanging base of labrum; antennal insertions moderately widely separated, supra-antennal carinae short, frontal carina medially obsolete; antenna inserted in small crescent-shaped socket flush with head capsule, with 11 antennomeres, bipectinate from antennomere IV; pronotum relatively short compared to elytra, wider than long, widest at posterior angles, posterior angles short, slightly divergent, non-carinate; lateral carina complete, strongly sinuate near posterior angle in lateral view; pronotosternal sutures very shortly excavate anteriorly, distinctly curved inwards; prosternum anteriorly truncate, without chin-piece; prosternal process rather long, slightly roundly emarginate behind procoxae, without subapical tooth; procoxal cavities moderately widely separated; protrochantin not visible; pro- and mesotrochanters elongate, slightly more than twice as long as wide; mesocoxal cavities narrowly separated, open to both mesanepisternum and mesepimeron; mesocoxae large, conical; metaventrite distinctly elongate; metacoxal plate rather narrow, gradually narrowed towards elytron; tarsomeres I–III apicoventrally with short membranous lamellae and IV with longer lamella; pretarsal claw simple; aedeagus with median lobe slightly surpassing tip of parameres, and with parameres having short and sharp subapical hook. For more details, see the description of *B. bipectinatus* sp. nov. below, Figs. 1–5, and Supplementary Videos 1–3.

*Etymology*. Derived from the words "Baltic" (referring to Baltic amber) and "*Elater*" (a genus name in Elateridae). Gender: masculine.

*Composition and distribution*. Only *B. bipectinatus* sp. nov. (Eocene Baltic amber).

### *Baltelater bipectinatus* sp. nov

(Figs. 1–5, Supplementary Videos 1–3).

urn:lsid:zoobank.org:act:5CCA61A9-9FE4-4922-A39C-A6467CE16099.

*Type material*. Holotype, adult male, "6685" (MAIG) (ex coll. Jonas Damzen, JDC 8374). A complete beetle is included in an elongate, transparent, yellow amber piece with dimensions of 48 × 19 × 3 mm, and preserved without supplementary fixation. Syninclusions consist of two different specimens of Nematocera (Diptera), many stellate Fagaceae trichomes, and several small gas vesicles.

*Type stratum and age*. Baltic amber from Eocene "Blue Earth" layers, predominantly Bartonian age (41.2–37.8 Ma) (for more details, see Material and methods).

*Type locality*. Baltic Sea coast, Yantarny settlement (formerly Palmnicken), Sambian (Samland) Peninsula, Kaliningrad Oblast, Russia.

*Diagnosis*. As for the genus.

*Description*. Body (Figs. 1, 2, Supplementary Video 1) 5.4 mm long, 1.6 mm wide, elongate, slightly convex, uniformly brown, covered with rather dense, semi-erect pubescence.

Head (Figs. 1–3) globular, deflexed, only partly retracted to prothorax so that eyes are entirely visible, including eyes distinctly wider than anterior margin of pronotum but slightly narrower than pronotum posteriorly; surface rather densely punctate; punctures large, coarse, deep, rounded to suboval, intervals between punctures usually less than a puncture diameter; densely covered with long, semi-erect pubescence. Frons apically slightly overhanging base of labrum; antennal insertions moderately widely separated, surface above them distinctly raised, forming short supra-antennal carinae, frontal carina not developed medially; frontoclypeal region slightly inflexed, transverse, about twice as wide as long (Fig. 3a–c). Eyes large, protuberant, entirely visible from above. Labrum rather small, transverse, convex, coarsely punctate, apically rounded. Mandible robust, rather broad and short, bidentate, lateral edge distinctly curved, region between apical teeth very short. Maxilla with palpus four-segmented, moderately long; palpomere I short, transverse; palpomere II elongate, about 1.7 times as long as wide; palpomere III slightly shorter than palpomere II; apical palpomere about as long as combined length of palpomeres II–III, about 1.7 times as long as wide at widest place, hatchet-like. Labium with palpus three-segmented, short; apical palpomere slightly longer than wide, hatchet-like. Antenna (Figs. 1–4, Supplementary Video 2) inserted in small crescent-shaped socket flush with head capsule, with 11 antennomeres, bipectinate from antennomere IV, stout, moderately long, slightly surpassing elytral humerus. Scape long, robust, notably thicker than remaining antennomeres; antennomeres II–III subequal in length, simple, approximately 1.4 times as long as wide, gradually widened apically; length ratio of antennomeres II–XI equal to 1.0 : 1.0 : 1.3 : 1.4 : 1.4 : 1.5 : 1.6 : 1.7 : 1.7 : 2.5; antennomeres IV–X slightly progressively increasing in length towards apex, bipectinate, with rami arising subbasally, slightly flattened to subcircular in cross-section, slightly longer than respective antennomere stems in antennomeres IV–VII, approximately as long as stem in antennomere VIII, and slightly shorter than respective stems in antennomeres IX–X (ramus/antennomere stem length ratio equals to 1.2 : 1.3 : 1.3 : 1.2 : 1.0 : 0.9 : 0.8); ultimate antennomere elongate, approximately three times as long as wide medially, widest at about middle, obliquely, distinctly narrowed apically, with apex narrowly rounded; all antennomeres moderately densely covered with semi-erect pubescence.

Pronotum (Figs. 2a, 3d) transverse, subtrapezoidal, widest at posterior angles, 1.1 times as wide as long anteriorly, 1.4 times as wide as long posteriorly, slightly convex, approximately 0.25 times as long as elytra. Anterior margin almost straight; anterior angles inconspicuous; lateral sides from dorsal view almost straight; posterior angles relatively short, sharp, slightly divergent, without apparent sublateral carina, apically narrowly rounded; posterior margin medially with shallow arcuate indentation, which is about as long as width of scutellar shield. Lateral carina distinct, complete, strongly sinuate near posterior angle in lateral view, in dorsal view visible only in posterior part, then hidden. Disc relatively sparsely punctate; punctures large, rounded, mostly separated by 0.5–2.0 times diameter of puncture, denser anteriorly, denser and smaller near posterior angles; surface densely covered with long, semi-erect pubescence. Hypomeron moderately densely covered with large, distinct punctures in anterior half and near pronotosternal sutures, almost smooth posteriorly near pronotal angles. Pronotosternal sutures very shortly excavate anteriorly, distinctly curved inwards. Prosternum (Figs. 1b, 2b, 3c) rather short, including prosternal process approximately 1.2 times as long as wide, in front of coxal cavities (i.e., excluding prosternal process) 0.6 times as long as wide, anteriorly truncate, without chin-piece. Prosternal process relatively long, slightly longer than prosternum in front of procoxal cavities, with sides subparallel-sided, slightly roundly emarginate behind procoxae, then abruptly narrowed towards apex, without apparent subapical tooth; surface uneven, coarsely punctate; apex narrowly rounded. Procoxal cavities subcircular, moderately widely separated; protrochantin not visible. Scutellar shield (Figs. 2a, 3d) about as long as wide, anterior margin almost straight; lateral sides and posterior margin rounded; surface covered with small punctures. Mesocoxal cavities narrowly separated, open to both mesanepisternum and mesepimeron; mesocoxae large, conical. Mesoventrite (Figs. 2b, 3c) transverse, slightly widened posteriorly; sides slightly sinuate; mesoventral process short, apically widely rounded; mesoventral cavity relatively shallow and with indistinct borders anteriorly, widened and with more distinct borders posteriorly. Metaventrite elongate, rather convex, moderately densely punctate; punctures smaller than those on prothorax; metanepisternum wider anteriorly and gradually narrowed towards posterior part. Metacoxal plate (Figs. 1b, 2b) rather narrow, abruptly emarginate near inner margin, only slightly, gradually narrowed towards elytron. Elytra (Fig. 2a,c) elongate, together approximately 2.5 times as long as wide, widest around apical 2/3; each elytron with striae formed by lines of large rounded punctures, which are separated by about diameter of a puncture; interstriae formed by smooth, convex ridges; pubescence dense, long, semi-erect to erect; elytral apices independently rounded. Hind wing well-developed, elongate. Leg slender, moderately long; pro- and mesotrochanters elongate, slightly more than twice as long wide; tarsomere I longest, about as long as tarsomeres II and III combined, tarsomere II longer than tarsomere III, tarsomere III longer than tarsomere IV, tarsomeres I–III ventrally with short lamellae, which are shortest on I, relatively short on II and slightly longer on III, tarsomere IV shortest, apicoventrally with well pronounced lamella, apical tarsomere elongate, slender; pubescence relatively sparse, semi-erect; pretarsal claw simple, moderately curved.

Abdomen (Figs. 1b, 2b,c) with five ventrites, first four of which seem to be more or less connate, with ventrite 5 free, lying on a different plane than ventrites 1–4; length ratio of ventrites 1–5 equal to 1.0 : 1.2 : 1.3 : 1.5 : 2.6; ventrite 1 with intercoxal process short, subtriangular; posterolateral corners of ventrite 1 produced and roundly acute, those of ventrites 2–4 less produced and more rectangular; surface of all ventrites covered with moderately large punctures and semi-erect pubescence, both denser at margins. Abdominal sternite IX only partly visible, elongate, gradually narrowed towards apex, apically narrowly rounded, apico-laterally finely punctate and with semi-erect pubescence. Aedeagus (Fig. 5, Supplementary Video 3) elongate, about 2.8 times as long as wide. Median lobe elongate, slightly surpassing tip of parameres, basally thicker, gradually narrowed towards apex, apically narrowly rounded and slightly curved from lateral view. Paramere elongate, with short and sharp subapical hook at about 1/4 paramere length when measured from apex; parameral apex with several long erect setae on outer margin. Phallobase not visible in the specimen.

Female and immature stages unknown.

*Etymology*. The specific name "bipectinatus" refers to the shape of the antennae (Fig. 4).

Biology. All known lissomine larvae are associated with rotten, decaying wood^41^, and therefore, we hypothesize that the larva of *B. bipectinatus* gen. et sp. nov. formed a part in the extinct amber forest ecosystem.

## Discussion

The finding of a new click-beetle genus in Baltic amber highlights the importance of X-ray micro-computed tomography for reconstruction of morphology in fossil organisms, especially in cases when opaque bubbles and the position or contraction of appendages obscures crucial diagnostic characters (Fig. 1). Further, this discovery confirms Baltic amber as an invaluable substance for understanding terrestrial palaeodiversity and evolution of various organisms as well as for reconstructing extinct Paleogene ecosystems. The fossils entombed in the relatively young Eocene European ambers are almost always classified within present-day suprageneric taxa (with some rare exceptions^21,62–65^), and approximately half of them also in extant genera^28^. Since newly described supraspecific taxa in Baltic amber are relatively less common than those in older ambers (e.g., Burmese, Lebanese), they represent very important evidence of rich Eocene palaeodiversity.

Regarding the Elateridae, exceptionally preserved fossils from Eocene ambers provide significant information about the evolution and diversification of this beetle family^2,6,28,53^. During the mid-Paleogene, all major present-day subfamilies already existed and had diversified^51^, which is documented by the fossil record of Agrypninae, Elaterinae, Dendrometrinae, Cardiophorinae, Negastriinae, Pityobiinae, and Omalisinae (Table 1), and further supported by recent dated molecular phylogenies, which date back the origin of the main Elateridae lineages to the Mesozoic^66–68^. However, although Elateridae are quite common in various Mesozoic and Cenozoic amber deposits^1,2,28,69^, their true diversity remains underexplored. This is mainly a result of their extremely homogeneous external appearance which is constrained by the development of the pro-mesothoracic clicking mechanism^41^. The problems surrounding a uniform morphology and multiple independent origins of various characters historically used for the delimitation of suprageneric taxa have greatly affected the classification of the group, which has been in a constant state of flux for both extinct and extant lineages^41–43,51,70^. This also affects the dating of the oldest known fossils for the subfamilies and hence for the whole family, since the placement of some earliest representatives of e.g., Protagrypninae (e.g., *Elaterophanes* Handlirsch, 1906), Dendrometrinae (*Alaodima* Dolin, 1980) and Cardiophorinae (*Mionelater* Becker, 1963) has recently been questioned^51^, and needs critical evaluation (Table 1). Most click-beetle higher taxa are not delimited by unique synapomorphies but rather by combinations of characters. These taxonomic definitions are often challenged by new discoveries and are limited to certain geographic regions^41–43^. Phylogenetic analyses based chiefly on morphology have shown that their results must be treated with caution^70^. Recent molecular analyses at least partly improved our understanding the phylogeny and classification of the present-day higher taxa^43,44,46,47^; however, this is not possible for fossils, for which we have only morphological data available. Therefore, for a systematic classification of the newly discovered *Baltelater* gen. nov., we have to rely completely on our knowledge of adult morphology of all extinct and present-day lineages. However, this is even more difficult when some taxa are defined by characters on the hind wing venation or other difficult-to-observe characters^43,70^.

**Table 1:** Overview of the Elateridae subfamilies with information on their earliest known fossils. For more details and references, see Kundrata et al.^48^.

Subfamily	Genus/species	Period/epoch, location	Age (Ma)
Agrypninae	Species of genera *Ageratus* Dolin, 1980, *Compsoderus* Dolin, 1980, *Litholacon* Dolin, 1980, *Cryptocardius* Dolin, 1980	Jurassic of Kazakhstan	166.1–157.3
Campyloxeninae	No fossils available		
Cardiophorinae	*Mionelater planatus* Becker, 1963	Miocene of Mexico	23.0–16.0
Dendrometrinae	*Alaodima grandis* Dolin, 1980	Jurassic of Kazakhstan	166.1–157.3
Elaterinae	Numerous undescribed genera and species	Cretaceous Burmese amber	99.6–93.5
Eudicronychinae	No fossils available		
Hemiopinae	No fossils available		
Lissominae	*Baltelater bipectinatus* gen. et sp. nov	Eocene Baltic amber	38.0–33.9
Morostominae	No fossils available		
Negastriinae	Species of genera *Ganestrius* Dolin, 1976 and *Protoquasimus* Dolin, 1976	Jurassic of Kazakhstan	166.1–157.3
Oestodinae	No fossils available		
Omalisinae	*Jantarokrama utilis* Kirejtshuk & Kovalev, 2015	Eocene Baltic amber	38.0–33.9
Parablacinae	No fossils available		
Physodactylinae	No fossils available		
Pityobiinae	*Cretopityobius pankowskiorum* Otto, 2019	Cretaceous Burmese amber	99.6–93.5
Protagrypninae	Two species of genus *Elaterophanes* Handlirsch, 1906	Triassic of the United Kingdom	208.5–201.3
Subprotelaterinae	No fossils available		
Tetralobinae	No fossils available		
Thylacosterninae	No fossils available		

*Baltelater* gen. nov. clearly belongs to Elateridae due to its exposed labrum, projecting posterior angles of pronotum, concealed protrochantins, well-developed metacoxal plates, and presumably connate first four ventrites^41,42^. Within Elateridae, many subfamilies can be easily ruled out as the close relatives of *Baltelater* gen. nov. by their rather divergent morphology^41–45,70,71^ (see Supplementary Text 1 for more detailed information). Instead, *Baltelater* gen. nov. clearly belongs to the subfamily Lissominae due to the anteriorly convex head, the medially incomplete frontal carina, the bidentate mandibles with a mandibular apex broad when viewed anteriorly (perpendicular to plane of movement), the antennal rami beginning on antennomere IV, the posterior margin of pronotum simple, without sublateral incisions, the metacoxal plates rather narrow, the tarsomeres I–IV each with a membranous lamella apico-ventrally, and the tarsal claws without basal setae on outer flat portion^41,47,61,71,72^. Lissominae are currently divided into two tribes—Lissomini, which are widely distributed, mainly in the tropics and subtropics, and Protelaterini, which occur in the south temperate regions (Australia, New Zealand, South America) and Asia from the Himalayas and China to the Greater Sunda Islands^43,47,61^. Lissomini have a typically compact habitus with the prothorax and elytra tightly joined together and not narrowed at the junction of prothorax and elytra. Further, they have characteristic deep antennal cavities lying beneath the hypomera, notably long and curved scape, well-developed and rounded chin-piece, very long trochanters, tibia which is slightly widened towards apex and weakly compressed laterally, tarsomeres I–IV each with a long membranous lamella (those on II–IV usually longer than the base of respective tarsomere), and styli of ovipositor absent^47,61^. Protelaterini are morphologically more diverse than Lissomini, and they differ from the latter in having the prothorax and elytra not tightly joined together and narrowed at the junction of prothorax and elytra, the scape more or less straight and only moderately long, the hypomeron without cavities for reception of antennae, the prosternal chin-piece usually short and truncate, the pro- and mesotrochanters less than twice as long as wide, the tibia subcylindrical and not compressed laterally, the membranous lamellae on tarsomeres less developed, and styli of ovipositor present, small, attached subapically or medially^47,61^ (for details on morphology of both Lissominae tribes, see Figs. 2–7 in a recent open access study^47^). The monophyly and internal relationships of Protelaterini are disputable^47,61,72^. Recent molecular phylogenetic analysis^47^ recovered *Austrelater* Calder & Lawrence, which is the most morphologically divergent genus within the Gondwanan Protelaterini, in a clade with *Senodonia* Laporte, 1838 and *Sossor* Candèze, 1883, which were traditionally placed near Dimini, far from the Lissominae. Unfortunately, the genera from South America and New Zealand were unavailable for the molecular analysis, mainly *Protelater* Sharp, 1877, which is the type genus of Protelaterini, and *Sphaenelater* Schwarz, 1902, which is the type genus of Sphaenelaterini, which is currently a synonym of Protelaterini^47,61^. Therefore, the classification of this group remains open to further study. *Baltelater* gen. nov. does not have the diagnostic characters of Lissomini but shares many morphological features with Protelaterini, e.g., the elongate body, the scape more or less straight and only moderately long, the prosternal chin-piece reduced, the hypomeron without deep antennal cavities, and the tibia subcylindrical and not compressed laterally. However, it differs from the extant Protelaterini genera in having the head exposed and not submerged in prothorax, and including eyes distinctly wider than the anterior margin of pronotum, the antenna bipectinate, the pro- and mesotrochanters slightly more than twice as long as wide (but still much shorter than in Lissomini), and abdominal ventrites 1–4 obviously not so tightly connected, with posterolateral corners more or less produced (Figs. 1–4, Supplementary Videos 1–3). The most morphologically similar extant genus is *Austrelater* from Australia, which, besides the typical Protelaterini characters, shares with *Baltelater* gen. nov. the large and strongly protuberant eyes, the similar shape of prothorax (including pronotum, prosternum, and pronotosternal sutures), metacoxal plates, and tarsomeres^41,47,72^ (Figs. 1–3; Fig. 6. in Kundrata et al. ^47^). Due to the unresolved internal relationships within Protelaterini^47^, the exact position of *Baltelater* gen. nov. remains to be determined until the study combining molecular markers and morphology is carried out using the broader sampling including the genera from New Zealand and South America.

The discovery of the first representative of Protelaterini in the European Eocene amber shed important light on the biogeography of the group. Protelaterini were traditionally considered as an exclusively southern hemisphere lineage known only from Australia, New Zealand, and the southern Neotropics^60,61^. Recently, Kundrata et al. ^47^ added *Senodonia*, *Sossor* and *Rostricephalus*, which expanded the distribution of this tribe into the Oriental and East Palearctic zoogeographical regions. Based on such distribution, Protelaterini may have been hypothesized as of a Gondwanan origin with a later expansion of their range to the eastern parts of Asia. However, our finding of *Baltelater* gen. nov. in the North European amber challenges this hypothesis. Similar cases in which the extant southern hemisphere taxon has its closest relatives in the Baltic amber include for example cyclaxyrid^9^ and some trogossitid beetles^73^, and Mantophasmatodea^74^.

The genus *Baltelater* gen. nov. proposed here is very distinctive due to its bipectinate antennae (Figs. 1–4). Bipectinate or biflabellate antennae are generally rare among Coleoptera but they occur in several lineages of Elateroidea, including Brachypsectridae, Eucnemidae, Lampyridae, Phengodidae, Rhagophthalmidae, and Elateridae^75^. Among Elateridae, these antennae can be found in several distantly related groups, and are composed of either 12 or, rarely, 11 antennomeres. Antennae with 12 antennomeres and with rami beginning on antennomere III were described in *Alaolacon* Candèze, 1865, *Euphemus* Laporte, 1838 (both Agrypninae: Hemirhipini), *Lacon bipectinatus* Riese, 1989 (Agrypninae: Agrypnini or Laconini), *Oplanischius* Chassain, 2010 (Elaterinae: Dicrepidiini) and *Didymolophus* Fairmaire, 1904 (Elaterinae: Aplastini or Morostominae), and those with 12 antennomeres and rami beginning on antennomere IV are known only in *Pityobius* LeConte, 1853 (Pityobiinae) and two species of *Anisomerus* Schwarz, 1897 (Eudicronychinae). Bipectinate antennae with 11 antennomeres and with rami beginning on antennomere III are known in *Mocquerysia* Fleutiaux, 1899 (Agrypninae: Hemirhipini) and *Sinoaplastinus* Schimmel, Platia et Tarnawski, 2008 (currently in Elaterinae: Aplastini but its position is dubious)^76^. All above-mentioned taxa are extant. The genus *Baltelater* gen. nov., however, has antennae with 11 antennomeres and with rami beginning on antennomere IV; this condition is known only in one other fossil genus, *Cretopityobius* Otto, 2019 (Pityobiinae) from Cretaceous Burmese amber^77^. This means that these two fossil genera bear a type of antenna which is not seen in any of the recent Elateridae. Additionally, bipectinate antennae are reported for the first time in a representative of Lissominae.

The peculiar morphology of the newly discovered elaterid genus *Baltelater* gen. nov. from Eocene Baltic amber significantly contributes to a better understanding of the palaeodiversity and evolution of the group. Although the systematics of Elateridae is far from fully understood and a reliable phylogenetic position of many lineages cannot be resolved by the use of only morphological characters due to abundant homoplasies, it is obvious that the study of fossils from various geological epochs may play an important role in the recognition of still-undescribed diversity and descriptions of new evolutionary lineages.

## Material and methods

This study is based on a specimen entombed in amber. The amber piece was polished by hand, allowing improved views of the included specimen, and it was not subjected to any supplemental fixation. Observations of this specimen were made using a Nikon SMZ 745 T stereomicroscope. The photographs were taken using a Canon 70D camera with a macro lens (Canon MPE-65 mm). Extended depth of field at high magnifications was achieved by combining multiple images from a range of focal planes using Helicon Focus v. 6.0.18 software. The X-ray micro-CT observations were conducted at Daugavpils University, Daugavpils, Latvia (DU) using a Zeiss Xradia 510 Versa system. Scans were performed with a polychromatic X-ray beam at an energy of 40 kV and power of 3 W. Sample to detector distance was set to 22.5 mm, and a source to sample distance of 24.2 mm was used. Tomographic slices were generated from 2401 rotational steps through 360-degrees of rotation, using a 4 × objective, and the exposure time during each projection was set to 6 s. Scanning parameters were set identical between two consecutive scans of the specimen in order to achieve smaller voxel resolution. Image stitching was carried out using automated reconstruction tool in Scout-and-Scan Control System (v14.0.14829.38124). Acquired images were binned (2 × 2 × 2) giving voxel size of 3.49 μm. Images were imported into Dragonfly Pro (ver. 4.1) software platform for interactive segmentation and 3D visualization. Volume renderings of X-ray microtomography of habitus, antennae and aedeagus are available in Supplementary Videos 1–3, respectively.

Body width of the examined specimen was measured at the widest part of the body, pronotal length at midline, and pronotal width at the widest part. Morphological terminology follows partly Calder^41^, Costa et al.^42^ and Kundrata et al.^47^, and the classification of Elateridae follows Kundrata et al.^43^. Although there are several hypotheses concerning the age of Baltic amber^36,37,39^, we follow the Middle Eocene (mostly Bartonian, 41.2–37.8 Ma) which was recently proposed for the extinct Central European resin-producing forests according to the stratigraphy of the Sambian amber deposits^40^. The material examined is deposited in the Museum of Amber Inclusions, University of Gdańsk, Poland (MAIG). For the morphological comparison of a new taxon with other Elateridae, we examined the representatives of all present-day suprageneric taxa deposited in various European and American museum and private collections, and additionally, we examined hundreds of fossil Elateridae from Baltic and Burmese amber deposited in the collections of the Paleontological Institute, Russian Academy of Sciences in Moscow, Russia (PIN), and collections of Robin Kundrata, Václav Dušánek (both Czech Republic), Andris Bukejs (Latvia), and Christel and Hans Werner Hoffeins (Germany). This included a majority of described species from ambers. For the remaining described fossil species, we used information from extensive literature, including the original descriptions (see Kundrata et al.^51^, and references therein). The ZooBank LSID number for this publication is: urn:lsid:zoobank.org:pub:B56223D7-57A5-4543-863D-5CF845DAED3A.

## Supplementary information

Supplementary Information 1.

Supplementary video 1

Supplementary video 2

Supplementary video 3

## Data Availability

All data needed to evaluate the conclusions in the paper are present in the paper and/or the Supplementary Materials. The original micro-CT scans and the videos of 3D volume renderings are deposited in Zenodo (original scans: https://doi.org/10.5281/zenodo.4061092; Supplementary Video 1: https://doi.org/10.5281/zenodo.4049708; Supplementary Video 2: https://doi.org/10.5281/zenodo.4054747; Supplementary Video 3: https://doi.org/10.5281/zenodo.4054755).
