# Supplementary material for: X-ray micro-computed tomography reveals a unique morphology in a new click-beetle (Coleoptera, Elateridae) from the Eocene Baltic amber
Source: Sci Rep. 2020 Nov 19;10:20158. doi: 10.1038/s41598-020-76908-3 (PMC7677381; doi:10.1038/s41598-020-76908-3)
Supplement: Supplementary file 1 — Supplementary Information 1. [file 41598_2020_76908_MOESM1_ESM.pdf]

X-ray micro-computed tomography reveals a unique morphology in a new click-beetle (Coleoptera, Elateridae) from the Eocene Baltic amber

Robin Kundrata[1,*], Andris Bukejs[2], Alexander S. Prosvirov[3], and Johana Hoffmannova[1]

[1]Department of Zoology, Faculty of Science, Palacky University, 17. listopadu 50, 771 46 Olomouc, Czech Republic
[2]Institute of Life Sciences and Technologies, Daugavpils University, Vienības 13, Daugavpils, LV-5401, Latvia
[3]Department of Entomology, Faculty of Biology, Moscow State University, Leninskie gory 1/12, 119234, Moscow, Russia
[*]robin.kundrata@upol.cz

List of Supplementary Materials

Supplementary Text. Morphological comparison of *Baltelater* gen. nov. with the Elateridae subfamilies except Lissominae.

Supplementary Video 1. Volume rendering of X-ray microtomography of the habitus of *Baltelater bipectinatus* gen. et sp. nov., holotype.

Supplementary Video 2. Volume rendering of X-ray microtomography of the antennae of *Baltelater bipectinatus* gen. et sp. nov., holotype.

Supplementary Video 3. Volume rendering of X-ray microtomography of the male genitalia of *Baltelater bipectinatus* gen. et sp. nov., holotype.

Supplementary Text

Morphological comparison of *Baltelater* gen. nov. with the Elateridae subfamilies except Lissominae

Within Elateridae, many subfamilies can be easily ruled out as the close relatives of *Baltelater* gen. nov. by their rather divergent morphology and therefore, only the most striking characteres are mentioned in the following overview. Unlike *Baltelater* gen. nov., Cardiophorinae have serrate antennae, mesocoxal cavities closed, and usually also a more convex pronotum with a reduced lateral carina, and an anteriorly emarginate scutellar shield. Similarly, Negastriinae have serrate antennae, a differently shaped prothorax, and mesocoxal cavities closed[1]. Parablacinae have a typically formed scutellar shield; either with the anterior margin rounded and gradually sloping downwards, or forming a cylindrical column with an oval-shaped apex; furthermore, they have serrate antennae[2]. Pityobiinae differ from *Baltelater* gen. nov. mainly in the antennae with 12 antennomeres, elongate posterior angles of pronotum, and an elongate prosternum with a well developed chin-piece. Hemiopinae and Oestodinae have serrate antennae and the posterior margin of the pronotum with typical sublateral incisions or carinae. Campyloxeninae have a complete frontal carina, serrate antennae, much longer posterior angles of the pronotum, and the prosternum distinctly elongate[3]. Eudicronychinae have a complete frontal carina, a distinctly longer prosternum, cleft claws, and modified male genitalia. Subprotelaterinae have a much more elongate prothorax and typical longitudinal grooves on the pronotal hypomera for reception of the antennae. Morostominae are an assemblage of morphologically divergent Madagascan genera which is in urgent need of revision, and the typical genera, such as *Morostoma* Candèze and *Diplophoenicus* Candèze, are easily recognized by their extremely elongate maxillary and labial palpi, and strongly flabellate antennae with 12 antennomeres, respectively. Physodactylinae have falcate mandibles and fossorial legs probably adapted to digging in the soil[4]. Omalisinae and Plastocerinae are morphologically modified due to neoteny, and except the softer cuticle they have a reduced prosternal process (from distinctly narrowed to completely reduced), and more than five abdominal ventrites[5,6]. Thylacosterninae have a completely different body shape along with the characteristic deep antennal cavities lying beneath the hypomera, and membranous tarsal lobes. Tetralobinae have a frontoclypeal region produced forward, the anterior part of the frons excavated and forming a thick projecting pad, the prosternum with a chin-piece, the metaventrite with the anterior margin elevated and V-

shaped (Piezophyllini) or metanepisternum large, wide, and forming about 1/4 of the metaventrite width (Tetralobini), tarsomeres I –IV apico-ventrally conspicuously lobed, and claws basally with setae[7]. Agrypninae form the second most diverse click-beetle subfamily and include lineages that can be usually easily differentiated from *Baltelater* gen. nov. based on distinct characters, including, e.g., the deeply grooved or anteriorly excavate pronotosternal sutures, body surface with scale-like setae, presence of luminescent organs, strongly modified tarsomeres, or a soft body with a reduced clicking mechanism and higher numbers of abdominal ventrites[4,5]. Other agrypnines, such as Hemirhipini, Oophorini or Pseudomelanactini, differ from *Baltelater* gen. nov. in, e.g., a usually more depressed body, complete frontal carina, smaller eyes, frontal margin of the pronotum more or less sinuate, anterior angles of the pronotum usually produced anteriorly, posterior angles usually with a sublateral carina, and the prosternum with a well developed chin-piece. Two other large subfamilies, Dendrometrinae and Elaterinae, together form more than half of the total click-beetle diversity, and although they are readily distinguished based on the larval morphology, they are often not easy to separate based on adult characters[2,4]. Dendrometrinae typically have a more-or-less depressed head with prognathous mouthparts, a more depressed body, an elongate and flattened prothorax, the frontal margin of the pronotum more-or-less sinuate, and the anterior angles of the pronotum more-or-less produced anteriorly. Most lineages share some additional characters which are not present in *Baltelater* gen. nov., such as posterior angles of pronotum elongate and with a sublateral carina, or prosternum with a distinct chin-piece. Most tribes of Elaterinae can easily be distinguished from *Baltelater* gen. nov. mainly by their globose head with a complete frontal carina, and usually also by the pronotum with sublateral carina(e) and the pronotosternal sutures double. The last two characters, in combination with others, are usually also present in the tribes which do not have a complete frontal carina, i.e., Elaterini, Agriotini, and Synaptini (and these have the frontal part of the head different from *Baltelater* gen. nov. in any case). The two remaining tribes, Cebrionini and Aplastini, are variously affected by neotenic development, often with a softer cuticle, modified morphology of the thorax, and a higher number of abdominal ventrites. Additionally, Cebrionini have distinctly falcate mandibles, the pro- and mesocoxal cavities almost contiguous, modified legs, and the parameres without a subapical hook. Aplastini resemble *Baltelater* gen. nov. in several characters, e.g., the large eyes, reduced anterior angles of the pronotum, and the prosternum with a reduced chin-piece; however, they differ considerably in the shape of head, the longer mandibes, the elongate prothorax, the posterior

angles of the pronotum with a sublateral carina, and the metacoxal plate distinctly narrowed towards the elytron.

References for Supplementary Text

1. Douglas, H. Phylogenetic relationships of Elateridae inferred from adult morphology, with special reference to the position of Cardiophorinae. *Zootaxa* **2900**, 1–45 (2011).
2. Calder, A. A. Click Beetles: Genera of Australian Elateridae (Coleoptera). Monographs on invertebrate taxonomy, Vol. 2. (CSIRO Publishing, Collingwood, 1996).
3. Arias-Bohart, E. T. *Malalcahuello ocaresi* gen. & sp. n. (Elateridae, Campyloxeninae). *ZooKeys* **508**, 1–13 (2015).
4. Costa, C., Lawrence, J. F. & Rosa, S. P. Elateridae Leach, 1815 in Coleoptera, Beetles; Volume 2: Morphology and Systematics (Elateroidea, Bostrichiformia, Cucujiformia partim) (eds. Leschen, R. A. B., Beutel, R. G. & Lawrence, J. F) in Handbook of Zoology, Arthropoda: Insecta (eds. Kristensen, N. P. & Beutel, R. G.) 75–103 (Berlin/New York: Walter de Gruyter GmbH & Co, 2010).
5. Kundrata, R. & Bocak, L. Molecular phylogeny reveals the gradual evolutionary transition to soft-bodiedness in click-beetles and identifies Sub-Saharan Africa as a cradle of diversity for Drilini (Coleoptera: Elateridae). *Zool. J. Linn. Soc.* **187**, 413–452 (2019).
6. Kusy, D., Motyka, M., Bocek, M., Vogler, A. P. & Bocak, L. Genome sequences identify three families of Coleoptera as morphologically derived click beetles (Elateridae). *Sci. Rep.* **8**, 17084 (2018).
7. Kundrata, R., Gunter, N. L., Janosikova, D. & Bocak, L. Molecular evidence for the subfamilial status of Tetralobinae (Coleoptera: Elateridae), with comments on parallel evolution of some phenotypic characters. *Arthropod Syst. Phyl.* **76**, 137–145 (2018).
